# The Transgenerational Consequences of the Interaction Between Humans and Molecules: Alcohol as a Cultural Artifact

**DOI:** 10.3389/fpsyg.2020.00061

**Published:** 2020-01-29

**Authors:** Alberto Granato

**Affiliations:** Department of Psychology, Catholic University of the Sacred Heart, Milan, Italy

**Keywords:** alcohol, addiction, intellectual disability, dopamine, development, plasticity, neurons, fetal alcohol spectrum disorders

With drinking, I cancel all my troubles;

What does it matter if I am poor?

*When I drink I am as rich as the rich Croesus*.

I really want to sing

*while I'm lying down, crowned with ivy*.

Here: I am the master of the world

*and if you want, o soldier*,

*goes to war too*.

*When you have fallen, pierced*,

I will be drunk, yes, but far more alive than you

(Anacreon, 5th cent. B.C.).

## Introduction

This paper aims to offer some insights on the concept of socio-materiality from the perspective of neuroscientific research. The example that will be proposed is that of the neuropsychological effects of alcohol abuse at both individual and intergenerational levels.

Alcohol consumption has always been a case of construction and use of cultural artifacts. Neuro-scientific studies related to the effects on the brain and behavior of a molecule (ethanol) transformed into consumer objects (alcohol) can profitably be integrated with psychosocial studies on the role of context and social practices in the predisposition to alcohol use/abuse to understand how the encounter with a socio-material element of the experience—alcohol—impacts on psychological development in the life span.

Specifically, with respect to the wide constellation of psychological phenomena related to the “galaxy” of alcohol consumption/abuse, some considerations will be offered on the fetal alcohol spectrum disorders (FASD). In fact, it is grounded in particular socio-cultural situations such as those typically connected to consumption during pregnancy and is one of the main causes of intellectual disability of the offspring; furthermore it also longitudinally predisposes to alcohol abuse in adolescence, enhancing the already strong social pushes to consumption of substances in that age of life also thanks to social alibis such as socializing disinhibition. In short, a vicious circle that must be interrupted, is rooted in the body, affects the mind, and costs to society. At the center of this vicious circle, a cultural artifact that, like many of the socio-material objects of our experience, loses its neutral character depending on the individual and social uses it allows (think of the pervasive debate on the various forms of technological addiction: Milani et al., 2018).

## The Burden of Early Alcohol Exposure

Unlike most drugs acting on our brain, ethanol, usually referred to as alcohol, when considered under the chemical point of view, is a quite simple molecule (CH_3_-CH_2_OH). This might explain why its well-known effect on the central nervous system has been long attributed to a non-specific interaction with the cell membrane of neurons. The neuroscientists now know that alcohol can interact in a specific way with the two main central neurotransmitters, modulating positively some GABA receptors and negatively some glutamate receptors (Lovinger et al., 1990; Weiner and Valenzuela, 2006). Furthermore, other neurotransmitter receptors, including those for the opioids and dopamine, as well as several voltage-gated ion channels, mediate the effects of alcohol on neurons (see Abrahao et al., 2017, for review). Therefore, despite its straight chemical structure, the interplay of alcohol and brain is definitely complex.

Drinking the first glass of wine during an adolescents' party can be an amazing experience, but for a few people the long-lasting outcomes of this encounter may eventually represent a dramatic devastation of their lives. Everything is made even more complicated by the fact that in most Western countries alcoholic beverages are legal, socially accepted, and belong to the consolidated culture. Moreover, new contexts, such as the social media, can boost alcohol consumption in young people (Hendriks et al., 2018).

For alcohol drinkers, the different periods of the lifespan matter, the most striking instance being maternal alcohol consumption during gestation. Drinking during pregnancy harms the brain development of the offspring and can result in FASD, one of the leading non-genetic causes of intellectual disability. The economic and social impact of alcohol misuse during pregnancy is dramatic and the annual cost for children affected by FASD exceeds that of other serious conditions, such as autism (Greenmyer et al., 2018).

Alcohol consumption during pregnancy is underreported in questionnaires (e.g., Morini et al., 2013) and FASD is underdiagnosed, especially in some countries (Vagnarelli et al., 2011) and in selected groups, such as adopted children (Bakhireva et al., 2018). Furthermore, although some authors think that prevention efforts should be devoted only to women with heavy drinking habits (Hatfield, 1985), there is compelling evidence, coming from both human and experimental studies, that even moderate or “social” maternal drinking can permanently impair offspring's cognitive functions (Olson et al., 1997; Valenzuela et al., 2012). Flak and coworkers, after carrying out a meta-analysis on the effects of different levels of prenatal alcohol exposure, conclude that “there is no known safe amount of alcohol to consume while pregnant” (Flak et al., 2014). Not to mention that many other substances of abuse, including cocaine, can negatively affect the brain development following exposure during critical gestational periods (reviewed in Ross et al., 2015). Finally, the co-exposure to more than one substance of abuse, such as alcohol and nicotine, can have a detrimental cumulative or synergic effect on the offspring's brain and cognitive function (e.g., Rivkin et al., 2008; Gautam et al., 2015). This is enough to warn the general public and the policy-maker about the risk of exposing the fetus to harmful molecules.

## A Priming Role of the Early Alcohol Experience?

**Figure 1:**
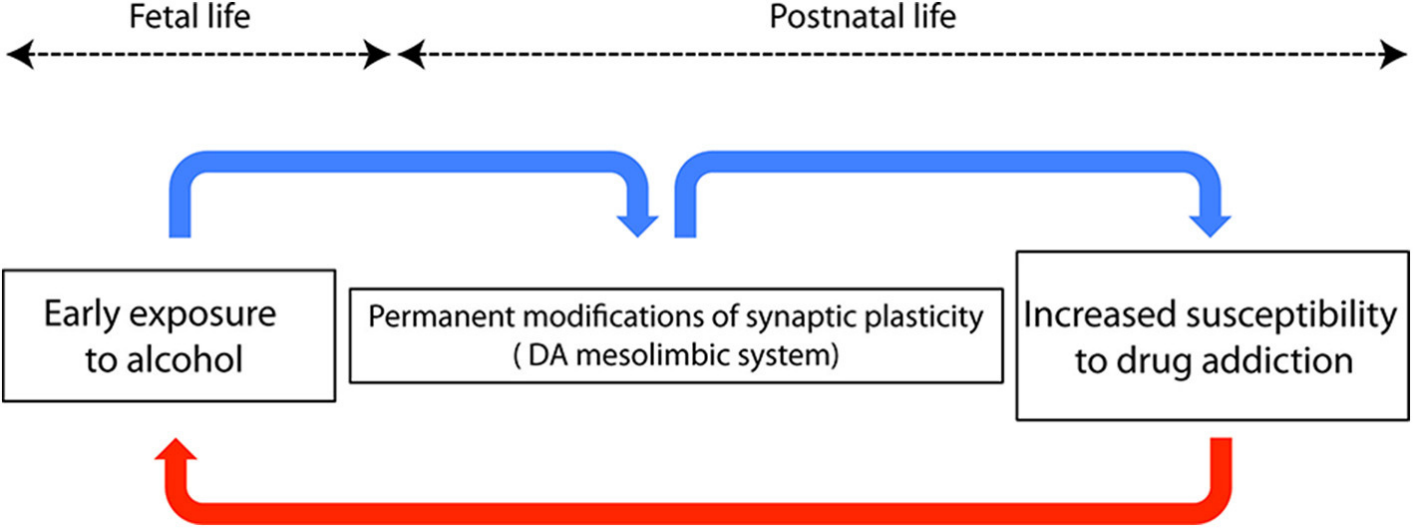
Schematic diagram showing a neurobiological hypothesis to explain the trangenerational self-sustaining circle of drug addiction. Blue arrows represent connections among events occurring in the same individual, while the red arrow represents transgenerational events.

But there's something else to be worried about. Adolescents and adults exposed to alcohol during fetal life show an increased risk of becoming addicted to alcohol and other drugs (e.g., Baer et al., 2003; Alati et al., 2006), thus perpetuating the damage in a kind of transgenerational self-sustaining vicious circle (Figure 1). One can argue that the higher risk of children of alcoholics is the consequence of several social, environmental, and genetic factors, not necessarily related to *in utero* exposure (Johnson and Leff, 1999). In addition, the intellectual disability *per se* might represent a risk factor for developing a substance abuse disorder (Carroll Chapman and Wu, 2012).

However, Yates et al. (1998) carried out a study on adoptees, ruling out several potential confounding variables, and concluded that fetal alcohol exposure increases the risk of later drug dependence. Many studies based on experimental reproductions of FASD in rodents helped to answer the question whether early alcohol is directly responsible for the development of drug seeking behavior during adulthood (see, Spear and Molina, 2005, for review). Recently, Wang et al. (2019), using conditioned place preference and self-administration paradigms^1^, demonstrated that prenatal ethanol increases the risk of psychostimulant addiction in adult rats. Therefore, a “priming” role exerted by early alcohol exposure should be taken into account when dealing with the higher risk of drug dependence in young adults affected by FASD. This is not surprising: even after experiencing something of apparent negligible significance, our neurons will never be the same anymore. In a word, neurons are plastic. The neurobiological counterpart of neuronal plasticity was first described by Bliss and Lomo in their seminal paper published in 1973 and is represented by a long lasting increase of synaptic efficacy following repetitive stimulation of glutamatergic hippocampal synapses (Bliss and Lomo, 1973). The observation of this phenomenon, referred to as long term potentiation (LTP), paved the way for the subsequent work on brain plasticity. Many studies pointed out that plasticity can be a good friend, but also a foe, since several neuropsychiatric disorders, including FASD, are characterized by a maladaptive plastic remodeling of neural circuits, and/or by a change of their plastic capacity (see Cohen et al., 2017). On this line of evidence, the permanent impairment of dendritic calcium electrogenesis observed in cortical neurons after early exposure to ethanol impacts synaptic plasticity, thus accounting for the FASD-related learning disabilities (Granato et al., 2012). Worth mentioning here, the neurobiological basis of addiction is currently considered a sort of “wrong” plasticity, or “excessive” memory of drug experience, occurring in the dopaminergic mesolimbic circuit, the reward processing area of the brain (see Kauer and Malenka, 2007). *In utero* exposure to alcohol triggers widespread death of neurons (Olney, 2014), whereas surviving cells undergo massive, possibly maladaptive, plastic adjustments, often caused by the same signaling cascade mediating apoptosis (Granato and Dering, 2018). The reward system itself is altered and its plastic responses are persistently modified, as demonstrated by the enhanced excitatory synaptic strength of dopaminergic neurons of the mesolimbic system in adult rats exposed to ethanol during prenatal life (Hausknecht et al., 2015). Considering the role of the dopaminergic system in the genesis of drug addiction, this finding can provide a mechanistic explanation for the increased risk of drug dependence in individuals who experienced an early exposure to ethanol (Figure 1). Other structures known to be involved in addictive behavior, such as the medial prefrontal cortex and the amygdala, are also affected by early exposure to alcohol and might contribute to generate drug dependence during later life (Baculis et al., 2015; Sharp, 2017; Cantacorps et al., 2019). Permanent consequences of early contacts with alcohol can be also explained by epigenetic mechanisms, i.e., by the long-lasting chemical modifications of DNA, some of which are known to be induced by ethanol (e.g., Mead and Sarkar, 2014; Cobben et al., 2019). Epigenetic modifications have also been demonstrated to be responsible for the transgenerational transmission of fetal alcohol effects through the male germline (Sarkar, 2016; Abbott et al., 2018), thus accounting for the paternal contribution to FASD (Abel, 2004).

Finally, prenatal alcohol can increase susceptibility to substance abuse via indirect mechanisms. For instance, FASD are characterized by a higher vulnerability to stress, depression/anxiety disorders (Hellemans et al., 2010), and aberrant pain sensitivity (Sanchez et al., 2017).

## Concluding Remarks

The key role played by the socioeconomic context and by education in the genesis of alcoholism cannot be underestimated (Boardman et al., 2001; Newton and Lee, 2019). Nevertheless, nature (i.e., neural circuits) and nurture (i.e., environmental context) are strictly interdependent, and can interact in such a way that the former is deeply modified by the latter. Eventually, in case of irreversible circuit changes, even the most refined attempts to improve the environmental conditions may result ineffective. This prompts the neuroscientist to search new therapeutic strategies to counteract the permanent and detrimental plastic changes induced by harmful environmental factors.

Considering alcohol as a cultural artifact with the profound implications for the body here described can lead to a profitable integration between the studies conducted by the neuroscientific and psychosocial perspectives, providing each of them with the opportunity to understand features that would escape from an unintegrated view.

We define alcohol abuse as a phenomenon at risk of intergenerational transmission. In this regard, the psychosocial view offers the possibility of understanding when and in which social and contextual framework the interaction with the molecule takes place; of this same interaction the neuroscientific view can provide a detailed comprehension of the specific mechanisms and their medium and long term consequences from the cerebral point of view.

## Author Contributions

The author confirms being the sole contributor of this work and has approved it for publication.

### Conflict of Interest

The author declares that the research was conducted in the absence of any commercial or financial relationships that could be construed as a potential conflict of interest.

## Footnotes

^1^Two paradigms largely used in experimental behavioral studies of addiction. The conditioned place preference is based on the observation that addicted animals (usually rodents) spend more time in one of two places in which they were given the drug during conditioning, compared to the other, in which they received only the vehicle (i.e., saline). In the self-administration protocol used in the cited study (Wang et al., 2019), animals were trained to press a lever to receive intravenous injection of the psychostimulant drug. Addicted animals self-administered more drug infusions than controls.
